# AI-enhanced diagnostic model for pulmonary nodule classification

**DOI:** 10.3389/fonc.2024.1417753

**Published:** 2024-08-30

**Authors:** Jifei Chen, Moyu Ming, Shuangping Huang, Xuan Wei, Jinyan Wu, Sufang Zhou, Zhougui Ling

**Affiliations:** ^1^ Department of Biochemistry and Molecular Biology, School of Basic Medicine, Guangxi Medical University, Key Laboratory of Biological Molecular Medicine Research (Guangxi Medical University), Education Department of Guangxi Zhuang Autonomous Region, Nanning, China; ^2^ Department of Pulmonary and Critical Care Medicine, The Fourth Affiliated Hospital of Guangxi Medical University, Liuzhou, China

**Keywords:** pulmonary nodule, diagnostic model, nomogram, DCA, lung cancer

## Abstract

**Background:**

The identification of benign and malignant pulmonary nodules (BPN and MPN) can significantly reduce mortality. However, a reliable and validated diagnostic model for clinical decision-making is still lacking.

**Methods:**

Enzyme-linked immunosorbent assay and electro chemiluminescent immunoassay were utilized to determine the serum concentrations of 7AABs (p53, GAGE7, PGP9.5, CAGE, MAGEA1, SOX2, GBU4-5), and 4TTMs (CYFR21, CEA, NSE and SCC) in 260 participants (72 BPNs and 188 early-stage MPNs), respectively. The malignancy probability was calculated using Artificial intelligence pulmonary nodule auxiliary diagnosis system, or Mayo model. Along with age, sex, smoking history and nodule size, 18 variables were enrolled for model development. Baseline comparison, univariate ROC analysis, variable correlation analysis, lasso regression, univariate and stepwise logistic regression, and decision curve analysis (DCA) was used to reduce and screen variables. A nomogram and DCA were built for model construction and clinical use. Training (60%) and validation (40%) cohorts were used to for model validation.

**Results:**

Age, CYFRA21_1, AI, PGP9.5, GAGE7, and GBU4_5 was screened out from 18 variables and utilized to establish the regression model for identifying BPN and early-stage MPN, as well as nomogram and DCA for clinical practical use. The AUC of the nomogram in the training and validation cohorts were 0.884 and 0.820, respectively. Moreover, the calibration curve showed high coherence between the predicted and actual probability.

**Conclusion:**

This diagnostic model and DCA could provide evidence for upgrading or maintaining the current clinical decision based on malignancy probability stratification. It enables low and moderate risk or ambiguous patients to benefit from more precise clinical decision stratification, more timely detection of malignant nodules, and early treatment.

## Introduction

According to the 2020 statistics, the global incidence and mortality rates of lung cancer are 32/100,000 and 45.4/100,000 respectively. The incidence and mortality of lung cancer exhibit significant international variation, primarily influenced by smoking patterns, gender, and economic development status. As the human development index increases, the incidence and mortality of lung cancer are projected to increase three to four times (1). In 2022, China reported approximately 48 million new cancer cases and over 3.2 million cancer-related deaths, with lung cancer ranking first in both morbidity and mortality (2). In the United States, approximately 48% of lung cancer patients were diagnosed with distant metastasis, and the 5-year survival rate for these patients is approximately 8%. Conversely, the 5-year survival rates for patients with localized and regional lung cancer are 62.8% and 34.8%, respectively (3). These findings underscore the significance of early diagnosis for pulmonary nodules.

To date, the most effective strategy for enhancing pulmonary nodule (PN) detection and significantly reducing lung cancer mortality is low-dose CT (LDCT) screening in asymptomatic individuals, as statistically validated by previous studies (4, 5). However, LDCT screening has a high false positive rate of 96.4% in differentiating between early lung cancer and benign nodules (6). This high rate of false positives, coupled with the potential for misdiagnosis (7), raises concerns about the cumulative radiation exposure from regular LDCT follow-up, which itself can become an independent risk factor for lung cancer (8). The differentiation between malignant pulmonary nodules (MPNs) and benign pulmonary nodules (BPNs) often relies more on the clinician’s experience or specific CT findings rather than on objective and reliable diagnostic methods (9). The Mayo Clinic model, which incorporates three clinical and three radiographic characteristics, has been extensively utilized for predicting the malignancy risk of pulmonary nodules since its introduction in 1997, demonstrating considerable diagnostic accuracy with an area under the curve (AUC) of 0.832 (10). However, it tends to underestimate the malignancy risk in patients deemed low-risk (11), and issues with misdiagnosis and overtreatment persist, particularly in patients with intermediate risk, in clinical settings (12).

Current diagnostic models for pulmonary nodules face several limitations. The Mayo Clinic model, which incorporates three clinical and three radiographic characteristics, has been extensively utilized for predicting the malignancy risk of pulmonary nodules since its introduction in 1997, demonstrating considerable diagnostic accuracy with an area under the curve (AUC) of 0.832. However, it tends to underestimate the malignancy risk in patients deemed low-risk, and issues with misdiagnosis and overtreatment persist, particularly in patients with intermediate risk, in clinical settings. Other existing models also struggle with accurately classifying nodules in the intermediate risk category, leading to potential delays in diagnosis or unnecessary invasive procedures. To address these limitations, there is a growing interest in integrating advanced technologies, such as artificial intelligence (AI), with traditional biomarker analysis. The rationale for this integration lies in the potential to combine the pattern recognition capabilities of AI with the molecular-level insights provided by immunoassays. AI algorithms can analyze complex imaging data to detect subtle features that may not be apparent to the human eye, while biomarkers can provide information about the underlying biological processes associated with malignancy. By combining these approaches, we aim to create a more comprehensive and accurate diagnostic model that can better stratify patients and guide clinical decision-making.

As a supplement to LDCT lung cancer screening, the utilization of serum biomarkers for the early detection of lung cancer has emerged as a burgeoning area of investigation (1, 13, 14). Our prior research has demonstrated that a combined diagnostic approach using a panel of four traditional tumor markers (4TTMs: CYFRA21, CEA, NSE, and SCC) yields high diagnostic efficiency, with an AUC of 0.854, a sensitivity of 89.4%, and a specificity of 61.9% in differentiating lung cancer from benign pulmonary diseases (15). Furthermore, a panel of seven autoantibodies (7AABs: p53, GAGE7, PGP9.5, CAGE, MAGEA1, SOX2, and GBU4-5) has demonstrated notable diagnostic performance, with an AUC of 0.742, a sensitivity of 67.5%, and a specificity of 74.1% in distinguishing early-stage lung cancer from BPN (9).

Recent advances in artificial intelligence (AI) have led to the development of CT image-based AI systems for detecting malignant pulmonary nodules (16). Massion et al. described a convolutional neural network (CNN)-trained diagnostic assistance system that has been increasingly utilized to reclassify indeterminate pulmonary nodules into low- or high-risk categories, achieving an AUC ranging from 0.835 to 0.919 (17). Furthermore, Wang et al. developed an automated triage system employing deep radiomics that demonstrated significant efficacy in the subtype classification of lung adenocarcinoma (AUC of 0.739-0.940) and in predicting patient survival (AUC of 0.846-0.937) (18). Additionally, Zhao et al. crafted a cross-modal 3D neural network that integrates CT images with prior clinical knowledge, which attained an AUC of 0.926 in predicting lymph node metastasis in clinical stage T1 lung adenocarcinoma (19). These findings underscore the immense potential and clinical value of AI in the diagnosis of lung cancer.

In this study, we gathered data on the risk assessment of pulmonary nodules as predicted by our institution’s Artificial intelligence pulmonary nodule auxiliary diagnosis system (AI), which is designed to assist in pulmonary nodule diagnosis. The AI system employs a convolutional neural network with deep learning algorithms that analyze CT images. We used a comprehensive set of variables, including the general condition of patients, MP outputs from the AI system, the Mayo Clinic model, and serum biomarkers (7AABs: p53, GAGE7, PGP9.5, CAGE, MAGEA1, SOX2, GBU4-5; and 4TTMs: CYFR21, CEA, NSE, SCC) to construct a diagnostic model. Our cohort comprised 188 patients with early-stage MPN and 72 patients with BPN, all of whom were recruited between 2017 and 2022. The objective was to enhance the differential diagnosis of pulmonary nodules and inform clinical decision-making processes.

## Materials and methods

### Patients and blood samples

This study, a diagnostic cohort test (Registration number: ChiCTR-DDD-17010378), was approved by the ethics committee of the Fourth Affiliated Hospital of Guangxi Medical University (Number KY2016208). Blood samples were collected from patients diagnosed with lung cancer (LC) or benign pulmonary nodules (BPN) via histopathology from January 2017 to May 2022, with informed, written consent obtained from each participant. Participant selection criteria included age 18 years or older, presence of a pulmonary nodule detected on CT scan, no prior history of lung cancer, no chemotherapy or radiotherapy prior to sample collection, and ability to provide informed consent. Exclusion criteria included pregnancy or breastfeeding, active infection or inflammation unrelated to the pulmonary nodule, and any condition that would interfere with the ability to comply with study procedures. A total of 260 participants meeting these criteria were enrolled in the study, including 188 patients with early-stage MPN and 72 patients with BPN. LC or malignant pulmonary nodule (MPN) was defined based on CT scans and verified by histopathology according to the World Health Organization Classification of Tumors (20). Pulmonary nodules (PN) were diagnosed by CT scans, and follow-up was performed in strict accordance with the Clinical Practice Consensus Guidelines (21). The patients’ blood samples were collected at initial diagnosis, with none of the LC patients having received preoperative chemotherapy or radiotherapy. PN is diagnosed clinically as a benign etiology if it accords with one of the following: (1) definitive pathologic diagnosis; (2) radiographic resolution; or (3) no evidence of growth according to CT scan for one year (22). Supernatants were obtained from blood samples through centrifugation at 3,000 g for 15 minutes at 4°C, immediately subpackaged, and then stored at -80°C until analyzed.

### Quantitation of 7AABs or 4TTMs panel in serum samples

The selection of the 7AABs and 4TTMs panels was based on their demonstrated relevance to lung cancer pathology and previous validation in clinical settings (23). The 7AABs panel (p53, GAGE7, PGP9.5, CAGE, MAGEA1, SOX2, and GBU4-5) was chosen based on its ability to detect autoantibodies against tumor-associated antigens that are often overexpressed or mutated in lung cancer. These autoantibodies can be detected in patient serum before clinical symptoms appear, making them valuable for early diagnosis (24). The 4TTMs panel (CYFR21, CEA, NSE, and SCC) consists of well-established tumor markers that have shown utility in lung cancer diagnosis and monitoring (15). The serum concentrations of the Seven-Autoantibody panel (7AABs), including p53, GAGE7, PGP9.5, CAGE, MAGEA1, SOX2, and GBU4-5, were quantified using Enzyme-Linked Immunosorbent Assay (ELISA) and a commercial AABs assay from Cancer Probe Biological Technology Co., Ltd, Hangzhou, China. The assay was performed in accordance with the manufacturer’s instructions and as previously outlined (25, 26). Specifically, samples and kit components were brought to room temperature and then diluted with phosphate-buffered saline. Subsequently, 50μL of diluted serum samples and standards were added to appropriate wells and incubated for one hour. Following three washes of the plate, 50μL of diluted secondary antibody anti-human IgG HRP was added to each well to bind the autoantibodies. The plate was washed three times and incubated for thirty minutes. The substrate was added, and the color development reaction was stopped after 15 minutes with 50μL of stop solution. The Optical Density (OD) at 450 nm was measured using Multiskan FC (Thermoscientific) within 30 minutes. Each sample was tested in duplicate. We utilized preset commercial cutoff values that offered maximum sensitivity with a fixed specificity of 90%, determined using a Monte Carlo direct search method (27). The serum concentration of the four traditional tumor markers panel, including CYFR21, CEA, NSE, and SCC, was quantified using an electro chemiluminescent immunoassay. All assays were conducted in accordance with instrument and reagent specifications, and cutoff values were established based on the manufacturers’ recommendations. The laboratory technicians were unaware of the patients’ identities, and the results were analyzed in a blinded manner by another investigator.

### AI (Artificial intelligence pulmonary nodule auxiliary diagnosis system) for predicting malignancy

The SHUKUN Science and Technology Co., Ltd. has developed an AI system that was officially approved and registered as a category III medical device by the State Drug Administration (registration number 20223210570, April 29, 2022). This system utilizes a sophisticated convolutional neural network (CNN) architecture specifically optimized for medical image analysis. The CNN consists of 18 convolutional layers, employing a ResNet-like structure with skip connections to preserve fine-grained details and mitigate the vanishing gradient problem. The network uses ReLU activation functions for non-linearity and includes batch normalization layers to stabilize training. The input layer accepts CT images in DICOM format, which undergo preprocessing including image normalization to a standard range of 0-1 and standardization to zero mean and unit variance. Data augmentation techniques, including random rotations (± 15°), translations (± 10% in x and y directions), and scaling (0.9-1.1), were employed to enhance model generalization. The model was trained on a dataset of 50,000 labeled CT images, comprising 30,000 benign and 20,000 malignant nodules, with a 80-10-10 split for training, validation, and testing. Hyperparameter tuning was performed using a combination of grid search and random search, optimizing for learning rate (final value: 0.001), batch size (32), and dropout rate (0.5). The model was trained using the Adam optimizer with a cosine annealing learning rate schedule over 100 epochs. To prevent overfitting, we employed L2 regularization (weight decay of 0.0001) and early stopping based on validation loss. The final layers of the network include a global average pooling layer followed by fully connected layers (1024, 512, and 256 nodes) with dropout. The output layer provides nodule characteristics (type, location, longest diameter, volume, average density) and a malignancy probability score. The model’s performance was validated on a separate test set of 5,000 images, achieving an AUC of 0.92 for malignancy prediction, demonstrating strong generalizability. To integrate biomarker data with imaging and clinical data, we employed a multi-modal fusion approach. The CNN-extracted features from the CT images are concatenated with the biomarker data (7AABs and 4TTMs) and clinical data (age, smoking history, etc.) in a fully connected layer. This combined feature vector then passes through additional fully connected layers (512 and 256 nodes) before the final classification layer. We used an attention mechanism to dynamically weight the importance of different data modalities, allowing the model to focus on the most relevant features for each case. This integration methodology significantly improved the model’s diagnostic accuracy, increasing the AUC from 0.92 (image-only model) to 0.96 (integrated model) on our test set. The integrated approach demonstrates superior performance in distinguishing between benign and malignant nodules, particularly in cases where imaging findings alone were ambiguous.


$$P(M|I,B,C)=\;\frac{1}{1+e^{-z}}$$

Where


$$z\;=\;\text{β}0\;+\;\sum_{i=1}^{n}\beta i\;fi\left(I\right)\;+\;\sum_{j=1}^{j=1}\gamma j\;Bj\;+\;\sum_{k=1}^{k=1}\delta k\;Ck$$

P(M|I,B,C) represents the probability of malignancy given the input data. I refer to CT image data, B denotes biomarker data (B_1, B_2,…, B_m), and C stands for clinical data (C_1, C_2,…, C_l). The term f fi(I) represents the i-th feature extracted from the CT image by the CNN. Bj is the j-th biomarker measurement, and Ck is the k-th clinical data point. The intercept term is represented by β0 , while βi are the coefficients for image features, γj are the coefficients for biomarker data, and δk are the coefficients for clinical data. The variables n, m, and l denote the number of image features, biomarkers, and clinical data points, respectively.

### Mayo (Mayo model) for predicting malignancy

The study also involved calculating the malignancy probability of the pulmonary nodules (PNs) using the Mayo predictive model, as defined by the following equations: probability (P) = ex/(1 + ex), x = −6.8272 + (0.0391 × age) + (0.7917 × smoking history) + (1.3388 × cancer history) + (0.1274 × diameter) + (1.0407 × spiculation) + (0.7838 × upper lobe), where e is the base of the natural logarithm. The variables for smoking history, cancer history, spiculation, and upper lobe can take on values of one for yes or zero for no. The diameter refers to the largest nodule measurement reported on the initial chest radiograph or CT scan (28).

### Statistical analysis

The study utilized R 4.3.2 (CBCgrps2.8 package) to conduct statistical description and bivariate statistical inference for each baseline variable. Additionally, the univariate ROC curve and AUC calculation were carried out using R 4.3.2, with the pROC and ggplot packages, respectively. Variable correlation analysis was performed using the corrplot package in R 4.3.2, while Lasso regression analysis was conducted with the glmnet package. Furthermore, univariate and multivariable logistic regression, as well as the creation of the Nomogram plot, were performed using R 4.3.2 and the rms package. Stepwise logistic regression was conducted with the MASS package, and the Frost plot was created using the forestmodel package. Finally, decision curve analysis (DCA) was implemented with the rmda package in R 4.3.2. The variable screening process included baseline comparison, univariate ROC analysis, variable correlation analysis, lasso regression, univariate and stepwise logistic regression, and decision curve analysis (DCA). Model fitting was conducted using multivariable logistic regression. The dataset was divided into training (70%) and validation (30%) cohorts for model validation. A nomogram and DCA were developed for clinical application.

## Results

### Baseline description of patients’ characteristics

**Figure 1:**
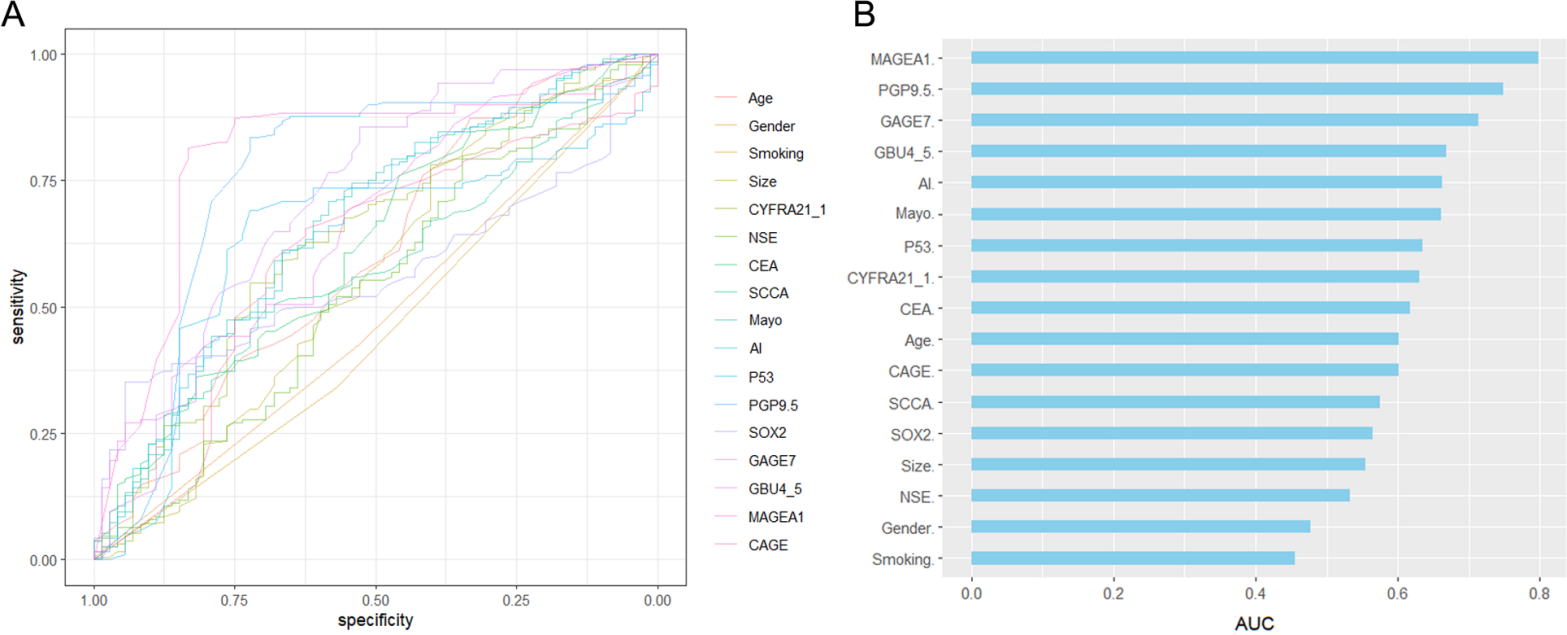
Receiver operating characteristic (ROC) curve analysis was conducted for each variable **(A)**, and the area under the ROC curve was used to sort the histogram **(B)**.

The statistical descriptions and bivariate statistical inferences of variables were summarized in **Table 1**. A total of 188 early-stage MPN (Malignant pulmonary nodules), 72 BPN (Benign pulmonary nodules) patients were included in the study. The etiologic diagnoses of the BPN group included inflammatory nodules (n=36), fungal infection (n=18), pulmonary tuberculosis (n=10), inflammatory pseudotumor (n=4) and hamartoma (n=4). There was no significant difference between BPN and early-stage MPN group in Gender, Smoking, and Size (*P=0.629, P=0.228, P=0.174*). However, significant statistical differences were found between the BPN and early-stage MPN groups in terms of Age (*P=0.005*), AI (*P<0.001*), Mayo (*P<0.001*), CEA (*P=0.003*), CYFRA21_1 (*P=0.001*), P53 (*P<0.001*), PGP9.5 (*P<0.001*), GAGE7 (*P<0.001*), GBU4_5 (*P<0.001*), MAGEA1 (*P<0.001*), and CAGE (*P=0.012*). These 11 variables were collectively defined as the Baseline panel. *Baseline panel*. Receiver operating characteristic curve (ROC) analysis was used to roughly evaluate the predictive value of each variable (**Figure 1A**), and the variables were sorted according to the area under the ROC curve (AUC) (**Figure 1B**).

**Table 1:** The statistical description and bivariate statistical inference of each variable.

Variables	BPN (n = 72)	Early-stage MPN (n = 188)	*P-value*
Age Mean ± SD	55.72 ± 11.95	60.28 ± 9.58	* * ***0.005***
Gender, n (%)			*0.629*
Female	38 (53)	108 (57)	
Male	34 (47)	79 (42)	
Smoking, n (%)			*0.228*
Never	41 (57)	124 (66)	
Ever/current	31 (43)	64 (34)	
Size(mm) Median (Q1-Q3)	15 (9.12-20)	16 (12.87-20.5)	*0.174*
Mayo (MP %) Median (Q1-Q3)	15.23 (9.39-30.77)	28.16 (15.53-50.48)	***< 0.001***
AI (MP %) Median (Q1-Q3)	15.94 (8.96-33.45)	31.21 (15.79-55.4)	***< 0.001***
4TTMs (ng/ml) Median (Q1-Q3)			
NSE	12.9 (10.79-14.79)	13.34 (11.41-15.06)	*0.418*
CEA	2.04 (1.4-3.13)	2.5 (1.8-4.35)	***0.003***
SCCA	0.89 (0.66-1.1)	0.97 (0.69-1.41)	*0.063*
CYFRA21_1	2.26 (1.85-2.78)	2.72 (2.1-3.27)	***0.001***
7AABs (U/ml) Median (Q1-Q3)			
P53	1.4 (0.9-4.43)	3.35 (1.78-5.55)	***< 0.001***
PGP9.5	0.4 (0.2-0.7)	3.25 (0.7-5.53)	***< 0.001***
SOX2	3.15 (1.5-4.75)	3.75 (1.2-12)	*0.104*
GAGE7	2.1 (1.5-3.8)	4.7 (2.28-6.93)	***< 0.001***
GBU4_5	1 (0.3-2.9)	2.4 (0.9-5.5)	***< 0.001***
MAGEA1	0.4 (0.1-0.5)	2.2 (1.15-4.75)	***< 0.001***
CAGE	0.8 (0.2-2.73)	2.2 (0.73-4.2)	***0.012***

AI, Artificial intelligence tool; Mayo, Mayo model; SD, standard deviation; Q1-Q3, first-third quartile, MPN, malignant pulmonary nodules; BPN, benign pulmonary nodules; MP, Malignant probability.The bold values indicate p < 0.05, which denotes statistically significant differences.

The italicization employed within the table is designed to adhere to the conventional standards for denoting statistical significance (P) and its corresponding values in scholarly articles published in reputable scientific journals. Should your specific journal prescribe alternative conventions, modifications to the use of italics or the adoption of a standard font may be implemented in alignment with the journal's prescribed guidelines.

### Screening workflow of the model variables

**Figure 2:**
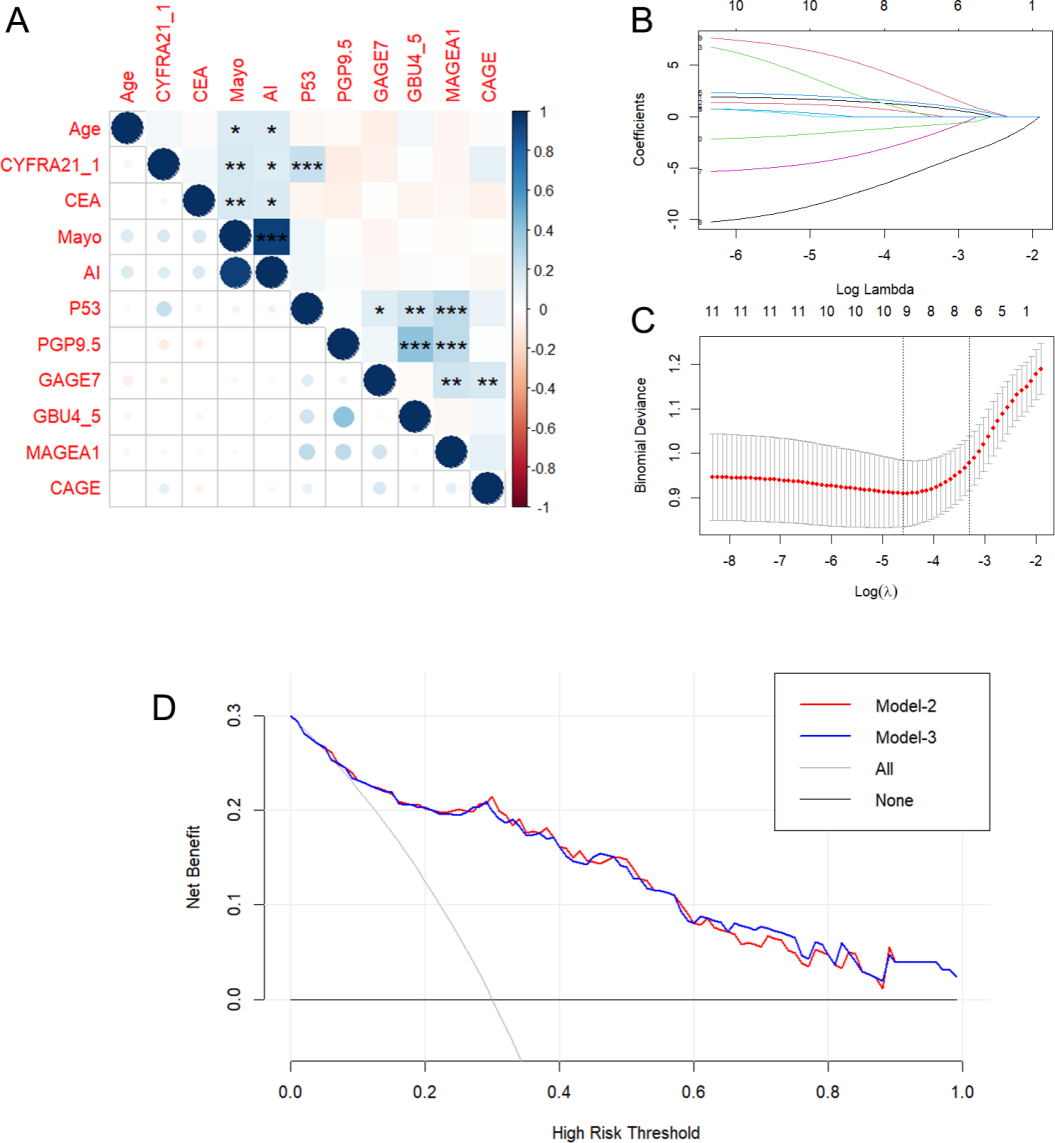
Pairwise correlation analysis **(A)** and Lasso regression **(B, C)** of the *Baseline panel*. And decision curve analysis **(D)** for Model-2 and Model-3. *p < 0.05, **p < 0.01, **p < 0.001, indicating levels of statistical significance.

The study began with a pairwise correlation analysis (**Figure 2A**) of the *Baseline panel*. The analysis revealed several significant correlations among the variables. Specifically, Age was found to have significant correlations with MAYO (*P<0.05*) and AI (*P<0.05*); CYFRA21_1 was correlated with MAYO (*P<0.01*), AI (*P<0.05*), and P53 (*P<0.001*); CEA was correlated with MAYO (*P<0.01*) and AI (*P<0.05*); P53 was correlated with PGP9.5 (*P<0.05*), GBU4_5 (*P<0.01*), and MAGEA1 (*P<0.001*); PGP9.5 was correlated with GBU4_5 (*P<0.001*) and MAGEA1 (*P<0.001*); and GAGE7 was correlated with MAGEA1 (*P<0.01*) and CAGE (*P<0.01*). These significant correlations indicate the need to simplify the variables. Next, univariate logistic regression of the Baseline panel (**Table 2**) identified Age (OR =1.05, 95% CI 1.02-1.08; *P=0.003*), Mayo (OR =1.02, 95% CI 1.10-1.04; *P=0.002*), AI (OR =1.03, 95% CI 1.01-1.04; *P=0.001*), GAGE7 (OR =0.83, 95% CI 0.75-0.91; *P<0.001*), GBU4_5 (OR =1.21, 95% CI 1.1-1.39; *P=0.001*), and MAGEA1 (OR =0.95, 95% CI 0.9-0.99; *P=0.019*) as valuable risk factors, which were then defined as the *Univariate panel*. Additionally, lasso regression (**Figures 2B, C**) of the Baseline panel suggested that the best variables for diagnostic model development were Age, CYFRA21_1, AI, PGP9.5, GAGE7, GBU4_5, and MAGEA1, which were defined as the *Lasso panel*. The Union panel, which was created by combining the Univariate panel and Lasso panel, included Age, CYFRA21_1, AI, Mayo, PGP9.5, GAGE7, GBU4_5, and MAGEA1. This Union panel was then used for the subsequent stepwise regression analysis (**Table 3**). The Akaike information criterion (AIC) of Model-2 and Model-3 were 229.27 and 228.51, respectively, and were chosen for the next decision curve analysis (**Figure 2D**). Model-3, with one less variable than Model-2, was found to have a high Net Benefit when the Risk Threshold was between 0.6-0.8.

**Table 2:** Univariate logistic regression of the *Baseline panel*.

Variables	OR	95%CI	*P-value*
Age	1.05	1.02-1.08	***0.003***
CYFRA21_1	1.35	1-1.92	*0.069*
CEA	1.11	1.01-1.29	*0.096*
Mayo	1.02	1.01-1.04	***0.002***
AI	1.03	1.01-1.04	***0.001***
P53	1.01	0.98-1.06	*0.431*
PGP9.5	0.96	0.91-1	*0.073*
GAGE7	0.83	0.75-0.91	***<0.001***
GBU4_5	1.21	1.1-1.39	***0.001***
MAGEA1	0.95	0.9-0.99	***0.019***
CAGE	0.99	0.92-1.08	*0.870*

OR, Odds Ratio; CI, confidence interval.The bold values indicate p < 0.05, which denotes statistically significant differences.

The italicization employed within the table is designed to adhere to the conventional standards for denoting statistical significance (P) and its corresponding values in scholarly articles published in reputable scientific journals. Should your specific journal prescribe alternative conventions, modifications to the use of italics or the adoption of a standard font may be implemented in alignment with the journal's prescribed guidelines.

**Table 3:** Stepwise regression analysis of the *Union panel*.

Models	Variables composition	AIC
Model-1	Age + CYFRA21_1 + AI + Mayo + PGP9.5 + GAGE7 + GBU4_5 + MAGEA1	230.98
Model-2	Age + CYFRA21_1 + AI + PGP9.5 + GAGE7 + GBU4_5 + MAGEA1	229.27
Model-3	Age + CYFRA21_1 + AI + PGP9.5 + GAGE7 + GBU4_5	228.51

AI, Artificial intelligence tool; Mayo, Mayo model; AIC, Akaike information criterion.

### Nomogram construction and validation

**Figure 3:**
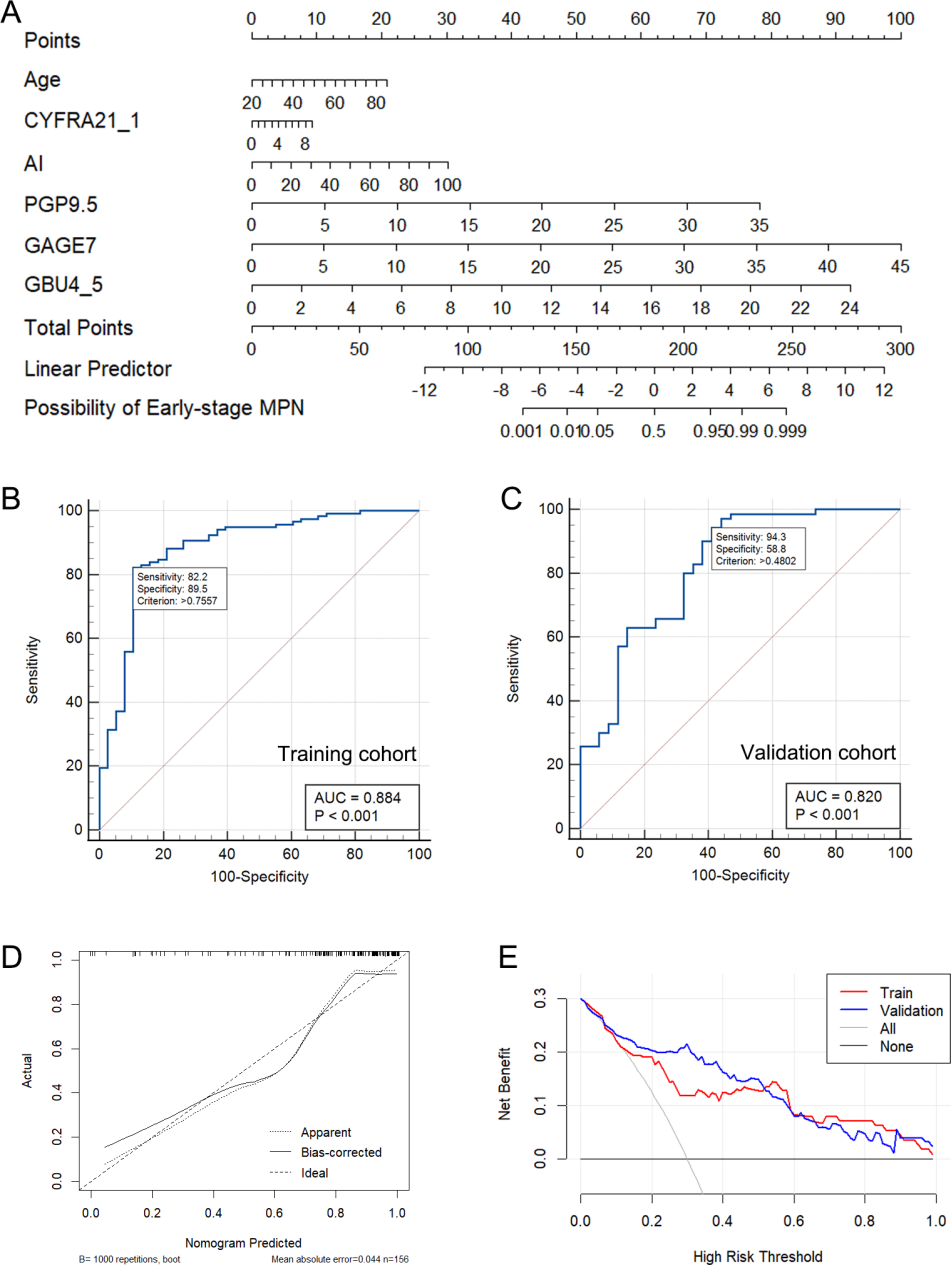
The nomogram constructed based on Model-3 **(A)**. The ROC curve of Model-3 in the training **(B)** and validation cohort **(C)**. The calibration plots of nomogram **(D)**. The DCA of the Model-3 **(E)**.

The 6 independent variates (Age + CYFRA21_1 + AI + PGP9.5 + GAGE7 + GBU4_5) in the Model-3 were incorporated to establish a nomogram for predicting Early-stage MPN probability (**Figure 3A**). A final logistic regression analysis, including these 6 variates, was conducted to demonstrate the odds ratio, 95%CI and *P-value* of each variable in the nomogram, and results were indicated in in **Table 4**. Subsequently, ROC curves were drawn to identify BPN and early-stage MPN in both the training (**Figure 3B**) and validation (**Figure 3C**) cohorts. In the training cohort, the AUC (95% CI, Sensitivity, Specificity) for predicting early-stage MPN was 0.884 (0.823-0.930, 82.2%, 89.5%), and in the validation cohort, it was 0.820 (0.726-0.883, 91.3%, 58.8%), indicating high discrimination of the mode. Furthermore, the performance of the nomogram was assessed by calibration plots (**Figure 3D**), which demonstrated great agreement between the predicted probability of early-stage MPN and actual observations, indicating good calibration of the model. Finally, decision curve analysis (DCA) was conducted according to the training and validation cohorts to guide clinical decision-making in practical use (**Figure 3E**).

**Table 4:** Coefficients, odds ratio, and 95% confidence intervals of the 6 predictors in logistic regression.

Variables	Coef	OR	95%CI	*P-value*
Age	0.03	1.03	1.00-1.06	*0.055*
AI	0.03	1.03	1.01-1.04	*0.001*
CYFRA21_1	0.22	1.24	0.91-1.69	*0.167*
GAGE7	-0.24	0.79	0.70-0.88	*<0.001*
GBU4_5	0.37	1.44	1.24-1.69	*<0.001*
PGP9.5	-0.18	0.83	0.77-0.90	*<0.001*

Coef, confidence interval; OR, Odds ratio; CI, Confidence Intervals confidence interval.

The italicization employed within the table is designed to adhere to the conventional standards for denoting statistical significance (P) and its corresponding values in scholarly articles published in reputable scientific journals. Should your specific journal prescribe alternative conventions, modifications to the use of italics or the adoption of a standard font may be implemented in alignment with the journal's prescribed guidelines.

## Discussion

Our study presents a novel diagnostic model that integrates AI-based image analysis with serum biomarkers for the classification of pulmonary nodules. This approach addresses several limitations of current diagnostic models and has the potential to significantly improve the accuracy of early lung cancer detection. The integration of AI and biomarker analysis allows for a more comprehensive assessment of pulmonary nodules, combining the pattern recognition capabilities of AI with the molecular insights provided by serum biomarkers.

Accurate differentiation between early-stage malignant pulmonary nodules (MPN) and benign pulmonary nodules (BPN) is crucial for the early diagnosis of lung cancer, significantly impacting the prognosis of lung cancer patients. Currently, the clinical management process for newly discovered solid or indeterminate nodules on CT is primarily based on nodule size (≤8 mm, 8-30mm) and Mayo malignancy stratification (5%, 5 - 65%,>65%), while also taking into account surgical or biopsy risk, the likelihood of active infection or inflammation, and the patient’s subjective willingness and compliance. The main clinical decisions include CT scan follow-up, PET imaging, non-surgical biopsy, and surgical resection (21, 29). However, due to the complexity of lung nodules and cancer itself, as well as the diverse clinical manifestations among individuals, there is still a significant number of misdiagnosed cases of early lung cancer. In particular, for patients at low and moderate risk, there remains ambiguity in selecting appropriate clinical decisions. Novel biomarkers have been developed to address these deficiencies in the early diagnosis of pulmonary nodules, such as the autoantibody group, circulating microRNA, small non-coding RNA (ncRNA), circulating tumor DNA, DNA methylation, complement fragments, blood protein profiles, and plasma lipid markers from liposome (13). Among these, the autoantibody panel EarlyCDT-Lung has been reported and validated as a tool to aid in the early detection of lung cancer (30, 31).

The present study utilized a derivation-validation methodological approach to develop and validate a diagnostic prediction model and to create a nomogram for differentiating between BPN and early-stage MPN. The model was constructed based on 18 variables, including age, sex, smoking history, nodule size, malignancy probability calculated by AI or Mayo, 7AABs (p53, GAGE7, PGP9.5, CAGE, MAGEA1, SOX2, GBU4-5), and 4TTMs (CYFR21, CEA, NSE, and SCC). These variables were collected from 72 BPNs and 188 early-stage MPNs at the Fourth Affiliated Hospital of Guangxi Medical University from January 2017 to May 2022.

Firstly, baseline description of patients’ characteristics was used for the statistical descriptions and bivariate statistical inferences of variables. The results showed that 11 variables (Age, AI, Mayo, CEA, CYFRA21_1, P53, PGP9.5, GAGE7, GBU4_5, MAGEA1, CAGE) exhibited significant statistical differences between BPN and early-stage MPN (**Table 1**). Next, univariate logistic regression analysis was used to identify risk factors for early-stage MPN in these 11 variables. The results indicated that only Age, AI, Mayo, GAGE7, GBU4_5, MAGEA1 were significant risk factors. Furthermore, Lasso regression was employed to eliminate significant correlations, resulting in the identification of 7 remaining variables (Age, CYFRA21_1, AI, PGP9.5, GAGE7, GBU4_5, MAGEA1). Additionally, a combination of the variables identified through univariate logistic regression and Lasso regression was used to prevent the erroneous deletion of effective variables, ultimately leaving a total of 8 variables (Age, CYFRA21_1, AI, Mayo, PGP9.5, GAGE7, GBU4_5, MAGEA1) for the final stepwise regression. The Akaike information criterion (AIC) was employed to evaluate the complexity and measure the goodness of fit of the statistical models (32). Finally, Model-2 (AIC=229.27) and Model-3 (AIC=228.51) (**Table 3**) were subjected to decision curve analysis (DCA) to determine whether the MAGEA1 variable should be removed from the model, as MAGEA1 achieved the largest AUC in single ROC analysis (**Figure 1B**). The performance of Model 2 and Model 3 in DCA was nearly identical. However, the net benefit of patient of Model-2 was slightly lower only when the risk threshold was between 0.6 and 0.8, but Model-3 ultimately lacked one predictive variable (**Figure 2D**).

The study developed an intuitive and user-friendly nomogram based on Model-3 to enhance its effectiveness in differentiating between BPN and early-stage MPN in clinical settings. The nomogram demonstrated a strong ability to predict early malignant pulmonary nodules in both the training and verification sets (**Figures 3B, C**). The calibration curve (**Figure 3D**) indicated good agreement between the predicted and actual probabilities. Importantly, the decision curve analysis (DCA) (**Figure 3E**) using the nomogram-derived risk probability can serve as a basis for adjusting or maintaining clinical decisions based on malignant probability (MP) stratification recommendations in current clinical practice. This allows for more precise clinical decision-making, timely detection of malignant nodules in low and moderate risk patients, and early treatment.

During the screening process, a high correlation was observed between the MP calculated by the AI pulmonary nodule auxiliary diagnosis system (AI) and the Mayo Model (Mayo) (**Figure 2A**). The AI currently in use at the Fourth Affiliated Hospital of Guangxi Medical University, which is based solely on CT images, does not demonstrate a clear advantage in the early diagnosis of pulmonary nodules compared to the traditional Mayo model (**Supplementary Figure S1**, AI vs. Mayo, P=0.956, AUC=0.662 vs. 0.661). Additionally, there were no significant differences in the composition of patients in the AI group compared to the Mayo group at all levels classified by MP (**Supplementary Table S1**). However, it is important to note that the AI system is capable of quickly locating the CT section of nodules, analyzing the length, type, and signs of nodules, and then calculating the MP. This greatly reduces the workload of radiologists, provides suggestions for diagnosis and treatment, and reduces misdiagnosis and missed diagnosis as previously reported (33, 34). As a result, the diagnosis and treatment of pulmonary nodules in our hospital have greatly benefited from the implementation of AI technology.

The developed model demonstrated high diagnostic accuracy, with AUC values of 0.884 and 0.820 in the training and validation cohorts, respectively. These results suggest that our model outperforms existing methods, including the Mayo Clinic model, in differentiating between benign and malignant pulmonary nodules. The nomogram and decision curve analysis (DCA) provide practical tools for clinical decision-making, potentially reducing misdiagnoses and improving early detection of lung cancer. However, it is important to acknowledge the limitations of our study. The sample size, particularly for benign pulmonary nodules, was relatively small, which may limit the generalizability of our findings. Additionally, our cohort was predominantly from a single ethnic group, and further validation in diverse populations is necessary. We also recognize that our model does not incorporate some of the latest biomarkers that have shown promise in lung cancer detection. This decision was made to focus on well-established markers and to create a model that is more readily applicable in current clinical settings.

Despite these limitations, our study represents a significant step forward in the development of personalized diagnostic tools for pulmonary nodule classification. The integration of AI and biomarker analysis offers a more nuanced approach to risk stratification, potentially allowing for more precise clinical decision-making, especially for patients in the intermediate risk category.

Future research should focus on validating this model in larger, more diverse cohorts and exploring the integration of additional biomarkers as they become validated. Furthermore, prospective studies evaluating the clinical impact of this model on patient outcomes and healthcare resource utilization would be valuable.

In conclusion, our AI-enhanced diagnostic model for pulmonary nodule classification demonstrates promising performance and has the potential to improve the accuracy of early lung cancer detection. By providing a more precise risk stratification, this model could help clinicians make more informed decisions about patient management, potentially leading to earlier detection and treatment of lung cancer while reducing unnecessary invasive procedures for benign nodules.

## Data Availability

The raw data supporting the conclusions of this article will be made available by the authors, without undue reservation.
