# Supplementary figures and images for: AI-enhanced diagnostic model for pulmonary nodule classification
Source: Front Oncol. 2024 Aug 30;14:1417753. doi: 10.3389/fonc.2024.1417753 (PMC11393475; doi:10.3389/fonc.2024.1417753)

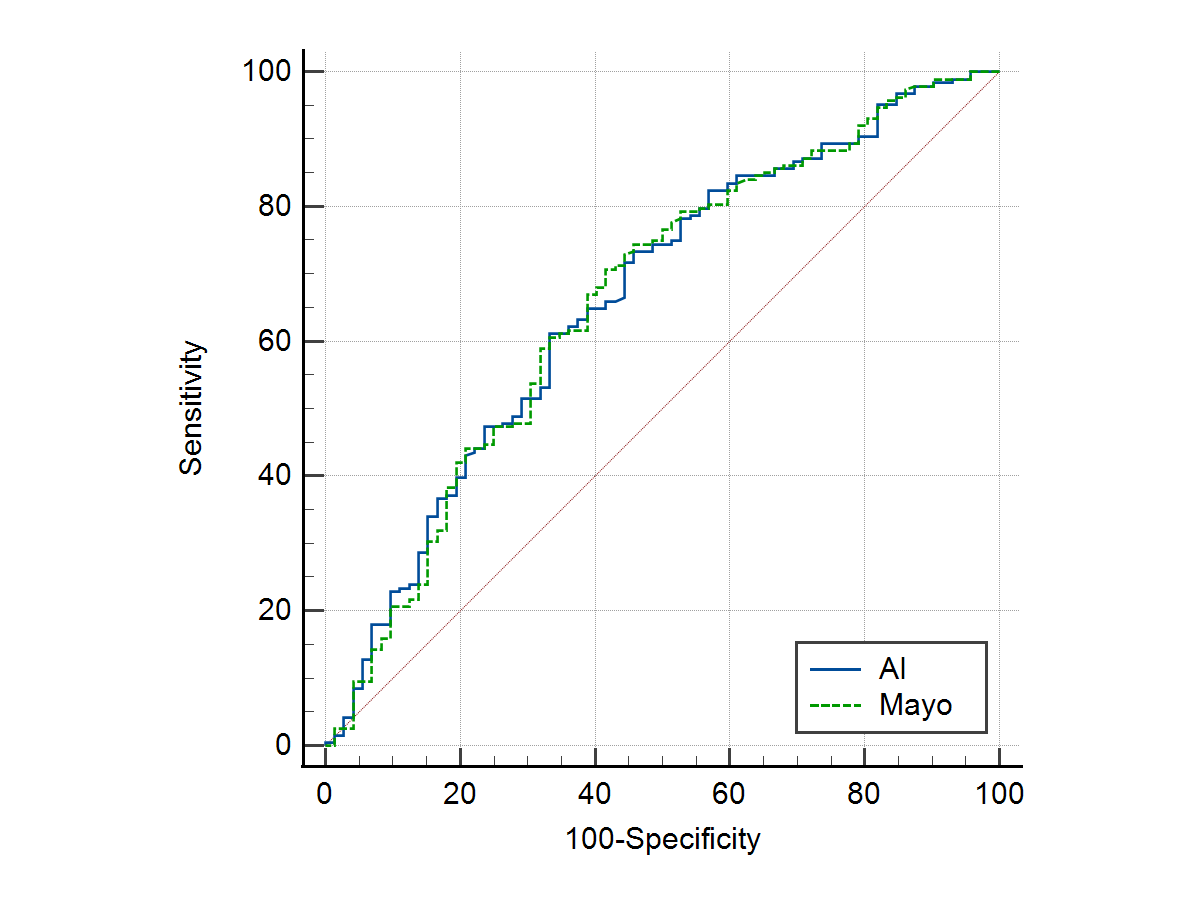

Supplement: Supplementary file 1 [file Image1.tiff]
