# Supplementary material for: AI-enhanced diagnostic model for pulmonary nodule classification
Source: Front Oncol. 2024 Aug 30;14:1417753. doi: 10.3389/fonc.2024.1417753 (PMC11393475; doi:10.3389/fonc.2024.1417753)
Supplement: Supplementary file 2 [file Table1.docx]

Table S1. Performance of the AI or Mayo in lung cancer detection

| **Parameters** | **BPN**  **(n=72)** | **Ⅰ-Ⅱ MPN**  **(n=188)** |
| --- | --- | --- |
| **Mayo**  n (%) |  |  |
| MP<5% | 13(18.1) *^**^* | 10(5.3) |
| 5≤MP≤65% | 54(75) ***^♀^*** | 148(78.7) |
| MP>65% | 5(6.9) ***^♀^*** | 30(16) |
| **AI**  n (%) |  |  |
| MP<5% | 13(18.1) *^**^* | 11(5.9) |
| 5≤MP≤65% | 54(75) ***^♀^*** | 155(82.4) |
| MP>65% | 5(6.9) ***^♀^*** | 22(11.7) |

AI, Artificial intelligence tool; Mayo, Mayo model; SD, standard deviation; MP, malignancy probability; MPN, malignant pulmonary nodules; BPN, benign pulmonary nodules. *^♀^>0.05, ^**^ <0.01*.

*.*
